# COVID-19: risk of ocular transmission in health care professionals

**DOI:** 10.47626/1679-4435-2021-598

**Published:** 2021-04-30

**Authors:** Alexis Galeno Matos, Ingrid Cavalcante Sarquis, Alana Andrade Neiva Santos, Leonardo Pereira Cabral

**Affiliations:** 1 Oftalmologia, Fundação Leiria de Andrade, Fortaleza, CE, Brazil; 2 Medicina do Trabalho, Associação Cearense de Medicina do Trabalho, Fortaleza, CE, Brazil

**Keywords:** COVID-19, eye, personal protective equipment, SARS, face shields

## Abstract

**Introduction:**

The current pandemic of severe acute respiratory symptom coronavirus 2 (SARS-CoV-2) has had a major impact on individuals’ lives. Social isolation and the use of personal protective equipment - the latter being especially important for health care workers - emerged as two of the main methods of preventing the spread of the disease. The eye can represent a source of transmission through contaminated tears, as well as a source of infection for respiratory droplets or aerosol particles, which may come into contact with the ocular surface and migrate to the lungs and other parts of the body.

**Objectives:**

To investigate the risk of ocular transmission through a literature review and identify ways of preventing it.

**Methods:**

A search of the scientific literature was conducted in the PubMed and Cochrane databases, using a combination of the following keywords: “COVID-19,” “eye,” “personal protective equipment,” “SARS-CoV-2,” “protective goggles,” “face shields,” and “workers’ health.”

**Results:**

The mechanisms of ocular transmission have not been fully elucidated, but studies have demonstrated the presence of viral RNA in the conjunctival sac and aerosolized secretions of contaminated patients; these droplets may come into contact with the eyes of uninfected bystanders, entering the respiratory system through the nose and gaining access to the lungs.

**Conclusions:**

Studies show that the virus can be effectively transmitted through the eyes, underscoring the importance of protective goggles for health care workers or potential transmitters of the virus, in addition to the need for additional education measures to encourage hand hygiene and discourage touching of the eyes.

## INTRODUCTION

Coronaviruses are enveloped, non-segmented, positive-strand RNA viruses belonging to the *Coronaviridae* (CoV) family.^1^ The current outbreak of severe acute respiratory syndrome coronavirus 2 (SARS-CoV-2) has been defined as a public health emergency of international concern by the World Health Organization (WHO).^2^ According to epidemiological data, the number of patients infected with novel coronavirus disease 2019 (COVID-19) in the first 2 months of the outbreak was nearly 10 times higher than that of individuals with severe acute respiratory syndrome (SARS), though the lethality of the disease was lower than observed in the 2003 outbreak of severe acute respiratory syndrome coronavirus (SARS-CoV-1) or the 2012 outbreak of Middle East respiratory syndrome coronavirus (MERS-CoV).^3^ SARS-CoV-2 uses angiotensin-converting enzyme 2 (ACE2) as a receptor to enter the host cells.^4^ ACE2 is a metallopeptidase expressed in epithelial cells of the respiratory system, eyes, small intestine enterocytes, and renal proximal tubule cells.^5^ It is responsible for the binding of the spike protein to the cell surface and mediates the entry of the virus into the host cell.^6^

The symptoms of COVID-19 include fever, dry cough, dyspnea, and ground-glass opacities in both lungs observed on computerized tomography.^1^ Yet some patients can also be asymptomatic while others present with gastrointestinal symptoms, such as diarrhea,^7^ and extrapulmonary manifestations, such as conjunctivitis, in the early stages of the disease.^8^ Severe cases have been mostly reported in individuals aged 60 and older, and tend to be associated with comorbidities such as cardiovascular illness, diabetes, chronic obstructive pulmonary disease, and arterial hypertension.^5^

Coronaviruses can produce several ocular manifestations, ranging from mild conditions of the anterior segment of the eye, such as conjunctivitis and anterior uveitis, to grave complications such as retinal detachment.^9^ In a previous study of COVID-19, 1/3 of patients with severe disease had ocular symptoms, which included conjunctival hyperemia, chemosis, epiphora, and increased ocular secretions.^10^ Given the high risk of contamination in health professionals who have direct contact with COVID-19 patients, the aim of this review was to assess the risk of ocular transmission and assess the importance of personal protection and hygiene in addressing this issue.

## METHODS

An integrative literature review was conducted to examine the research on ocular transmission of the coronavirus. Scientific articles were retrieved by searching the PubMed and Cochrane databases. The search was conducted using a combination of the following keywords: “COVID-19,” “eye,” “personal protective equipment,” “SARS-CoV-2,” “protective goggles,” “face shields,” and “workers’ health.” The articles retrieved in the search were reviewed, and their main findings were described in this study. The selection process involved title and abstract screening, followed by the retrieval of full-text articles. Studies whose abstracts did not address the topic of this review were excluded.

The initial search yielded 268 studies. After excluding duplicate articles and studies with overlapping samples, 28 articles remained in the review. After titles and abstracts were screened, relevant studies with full-text availability were selected for inclusion. The present study summarizes the main findings of the literature review.

## RESULTS AND DISCUSSION

According to the studies reviewed, the infection is transmitted through mucous membranes; aerosolized particles in the air; direct or indirect contact (for instance, touching the face or eyes); and by the fecal-oral route.^11^ The eyes can therefore be a source of infection and site of transmission.^12^ In a recent study, Hui et al.^13^ demonstrated that SARS-CoV-2 could infect conjunctival cell cultures and undergo productive replication, which suggests that the ocular route may be a pathway of transmission of COVID-19.^12,13^ The receptors found in the eyes and lungs are described in Table 1.^14^

**Table 1:** Comparison of SARS-CoV, SARS-CoV-2 and MERS-CoV receptors on the ocular surface and the lungs^14^

Coronaviridae	Receptor	Role in human CoV disease	Ocular surface (cell types)	Lungs
SARS-CoV SARS-CoV-2	ACE2	Host cell receptor, crucial for infection	+ (fibroblasts and conjunctival epithelium; corneal epithelium)	+ (airway epithelium)
	TMPRSS2	Primes spike protein for binding to ACE2	-	+ (airway epithelium)
	CD209	Cell-to-cell viral transfer	+ (corneal dendritic cells)	+ (alveolar macrophages)
MERS-CoV	CD26	Host cell receptor, crucial for infection	+/- (conjunctival vascular endothelium)	+ (pulmonary epithelium and endothelium)
	CD66e	Co-receptor	+/- (palpebral conjunctiva)	+ (bronchial and alveolar epithelium)

- = no reports of the receptor on the ocular surface; + = receptor has been found; +/- = found only in non-virally-mediated inflamed tissue; MERS-CoV = Middle East respiratory syndrome coronavirus; SARS-CoV = severe acute respiratory syndrome coronavirus; SARS-Cov-2 = severe acute respiratory syndrome coronavirus 2.

The coronavirus was detected in the tears of patients with SARS, although the detection rate of SARS-CoV-2 in the conjunctival sac was low.^15^ The infectiousness of tears and conjunctival secretions is difficult to evaluate due to the low sensitivity of RT-PCR testing.^5^ While tears also contain antimicrobial agents such as lactoferrin and secretory IgA, the constant rinsing of the eye may transport the virus from the ocular surface to the nasal cavity through the nasolacrimal duct.^16^ The outer lipid layer of the lacrimal film also increases resistance to pathogen invasion. This lipid layer is not present in nasal and respiratory mucosa,^17^ and as such, if contaminated aerosol particles are deposited on the surface of the eye, they may enter the nasolacrimal duct and access the lungs and other organs.^6,18^

Studies have identified viral RNA in the conjunctival sac of contaminated patients, underscoring the need for health care workers to wear protective goggles when in contact with patients.^19^ There have been reports of health care professionals infected with SARS-CoV-2 who did not wear protective goggles and had conjunctivitis as the first symptom of the disease.^9,20^ A previous study has also found that health professionals who come into contact with eye secretions without the necessary protections are at higher risk of SARS infection.^20^ SARS-CoVs have been found to spread by both direct and indirect contact with the ocular mucosa.^6^

Health care professionals have some of the highest levels of stress among all occupations. Individuals working in health care services, especially inpatient units, are routinely exposed to several physical and psychological demands.^21^ A well-equipped workforce in good physical and mental health is crucial for the effective management of COVID-19 at a national level.^22^ A report from the Centers for Disease Control and Prevention (CDC) showed that 3% of individuals who contracted COVID-19 were health care workers, and in a sample of 1,423 patients, which included health care professionals, 780 (55%) reported that their only contact with sick patients in the 14 days prior to disease onset occurred in health care settings. Among health professionals with information available regarding age and health outcome, while 90% of these professionals were not hospitalized, 8 to 10% were admitted to hospitals, 2 to 5% were treated in intensive care units (ICUs), and 0.2 to 0.6% died.^23^ In Italy, 20% of working health professionals have been infected with COVID-19.^24^

As the pandemic progresses, access to personal protective equipment (PPE) for front-line workers emerges as an important concern.^24^ Health care workers (physicians, nurses, physical therapists, etc.) must be careful when performing ophthalmologic examinations, orotracheal intubation and secretion aspiration or collecting swab samples for RT-PCR; these procedures require close proximity to patients’ faces, and as such, professionals must take the necessary precautions to prevent contamination and the spread of the disease (Table 2).^25,26^ Eye infections can be caused by aerosol exposure or self-inoculation. In a meta-analysis of 44 studies, Chu et al.^27^ reported that the use of eye protection led to a 78% reduction (adjusted odds ratio [aOR] 0.22; 95% confidence interval [95%CI] 0.12-0.39) in infections.^27^

**Table 2:** Recommended personal protective equipment for workers during the COVID-19 outbreak depending on place of work and occupation

Place of work	Occupation	Activities performed	Type of PPE
Bedside	Health care workers	Providing direct care to patients with COVID-19	Medical mask, boots, gloves, eye protection
		Aerosol-generating procedures performed on patients with COVID-19	N95, FFP2 or equivalent respirator; gown; gloves; eye protection
	Cleaning staff	Entering the room of patients with COVID-19	Medical mask, gown, heavy duty gloves, eye protection (if there is risk of splashing of organic material or chemicals), boots or closed work shoes
Laboratory	Laboratory technician	Manipulation of respiratory samples	Medical mask, gown, gloves and eye protection (if risk of splashing)
Consultation room	Health care professionals	Physical examination of patients with respiratory symptoms	Medical mask, gown, gloves, eye protection
	Cleaning staff	After and between appointments with patients with respiratory problems and symptoms	Medical mask, gown, heavy duty gloves, eye protection (if there is risk of splashing of organic material or chemicals), boots or closed work shoes
Home	Health care professionals	Providing direct care or assistance to patient with COVID-19 at home	Medical mask, gown, gloves, eye protection
Ambulance or transfer vehicle	Health care professionals	Transporting patients with suspected COVID-19 to referral centers or health care services	Medical mask, gown, gloves, eye protection
	Driver	Assisting with loading and unloading patients with suspected COVID-19	Medical mask, gown, gloves, eye protection
	Cleaning staff	Cleaning after and between transport of patients with suspected COVID-19 to referral health center	Medical mask, gown, heavy duty gloves, eye protection (if there is risk of splashing of organic material or chemicals), boots or closed work shoes

Source: adapted from "Rational use of personal protective equipment for coronavirus disease 2019 (COVID-19)," published by the World Health Organization.^28^ COVID-19 = coronavirus disease 2019; PPE = personal protective equipment.

Protective eyewear is recommended as the primary means of protection for the eyes, as it reduces aerosol exposure. In a previous study involving simulated cough droplets with a median diameter of 8.5 µm, face shields were able to reduce inhalation exposure by 96% at a distance of 46 cm. However, the device was only able to block 68% of particles with a diameter of 3.4 µm, and after a period of 1 to 30 minutes, the effectiveness of the face shield decreased to 23%. These findings suggest that face shields provide insufficient protection against small aerosol particles, and should not be used as the sole means of protection by professionals exposed to potential contamination for long periods of time.^29^

In conclusion, the COVID-19 pandemic is likely to affect current perspectives on the effectiveness of PPE, hand hygiene and avoiding contact with the eyes as methods of containing epidemics. The eye can represent a source of transmission through infected tears, as well as a pathway for infection by respiratory droplets or aerosol particles that come into contact with the conjunctiva Workers in several occupations may be vulnerable to ocular infection by SARS-CoV-2, especially in situations where people are not wearing protective masks.

The idea that droplets of saliva can transmit infection by coming into contact with the eye would support the implementation of educational initiatives to encourage the use of protective eyewear, with or without facial protection, in health care and other occupations. Professionals involved in emergency services (such as ambulance drivers) as well as cleaning and transportation staff should also be considered, as they may be exposed to contamination via the ocular route.

Professional training on preventive measures such as hand hygiene and the use of PPE can also contribute to the reduction in transmission rates in health care services, and may be especially important for workers with risk factors or comorbidities. It is crucial that every effort is made to ensure the health and safety of health care professionals.
